# Broadband Polarization-Insensitive Tunable Terahertz Metamaterial Absorber Based on an Asymmetric Graphene Structure

**DOI:** 10.3390/nano16090502

**Published:** 2026-04-22

**Authors:** Ahmed Ali, Sulaiman Al-Sowayan, Waleed Shihzad, Asrafali Barkathulla, Zaid Ahmed Shamsan, Majeed A. S. Alkanhal, Yosef T. Aladadi

**Affiliations:** 1 College of Physics and Optoelectronic Engineering, Shenzhen University, Shenzhen 518060, China; 2 Department of Electrical Engineering, College of Engineering, Imam Mohammad Ibn Saud Islamic University (IMSIU), Riyadh 11432, Saudi Arabia; sssowayan@imamu.edu.sa (S.A.-S.);; 3 Telecommunication Engineering Department, University of Engineering and Technology, Mardan 23200, Pakistan; 4 THz Technical Research Center of Shenzhen University, Shenzhen Key Laboratory of Micro-Nano Photonic Information Technology, Key Laboratory of Optoelectronic Devices and Systems of Ministry of Education and Guangdong Province, College of Physics and Optoelectronic Engineering, Shenzhen University, Shenzhen 518060, China; 5 Department of Electrical Engineering, College of Engineering, King Saud University, Riyadh 11421, Saudi Arabia

**Keywords:** metamaterial absorber, graphene, polarization insensitivity, tunable, impedance matching, terahertz applications

## Abstract

A graphene-based tunable broad-band terahertz (THz) metamaterial absorber is presented, exhibiting strong and stable absorption across a wide frequency range. The device employs an ultra-thin three-layer structure consisting of a metallic reflector, a dielectric spacer, and a patterned graphene metasurface with an asymmetric geometry. Through optimized structural parameters, the absorber achieves broad-band absorption exceeding 90% between 2.45 THz and 6.11 THz with a bandwidth of 3.66 THz, featuring three distinct resonant frequencies at 2.764 THz, 3.534 THz, and 5.41 THz, corresponding to peak absorption efficiencies of 97.26%, 96.96%, and 99.90%, respectively. Impedance matching and electric field analyses confirm that the enhanced absorption arises from the strong coupling of electric and magnetic resonances within the multilayer structure. Moreover, the absorber exhibits polarization-insensitive behavior under varying polarization angles and maintains high absorption stability for both TE and TM modes up to an incident angle of 60°, as verified by simulation results, and allows dynamic tunability through Fermi-level modulation. These characteristics highlight the absorber’s potential for advanced THz imaging, sensing, and stealth applications.

## 1. Introduction

Terahertz waves typically have a frequency range of 0.1–10 THz, and their corresponding wavelength falls between 30 μm and 3 mm [1,2,3]. Compared to other electromagnetic wave frequencies, terahertz waves have numerous special characteristics. Their frequency is lower than infrared and higher than microwaves, with an energy magnitude in between that of electrons and photons. Excellent application opportunities exist in the domains of communications, radar, electronic countermeasures, electromagnetic weaponry, medical imaging, and security inspections due to these special characteristics [4,5,6]. Researchers worldwide recognize the significant scientific potential of terahertz technology, which is currently the subject of ongoing research and development. The topic is growing in popularity, particularly in the military sector, where stealth is necessary [7]. One of the most popular study topics among scientists is how to accomplish sustained and effective terahertz absorption.

Metamaterial absorbers (MMAs) represent one of the most significant applications of metamaterials: artificial electromagnetic (EM) materials composed of periodic subwavelength microstructures that exhibit properties unattainable in conventional materials [8,9]. These absorbers effectively capture incident electromagnetic energy, convert it into heat, and thereby suppress reflection back to the source. MMAs have been demonstrated across various frequency ranges, including the terahertz (THz) [10], microwave [11], millimeter-wave [12], and near-infrared [13] bands. In particular, THz-operating MMAs have garnered considerable research attention due to their promising applications in wireless communication, solar energy harvesting, imaging, thermal emission control, detection, cloaking, and stealth technologies [14,15,16,17,18,19,20]. For instance, Landy et al. first demonstrated a perfect single-band metamaterial absorber (MMA) through simulation and experiment in 2008 [21]. Since then, researchers have developed MMAs exhibiting multi-band absorption characteristics, including penta-band [22], hexa-band [23], seven-band [24], and even eight-band [25] responses. However, due to the fixed electrical conductivity and rigid physical structure of metallic components after fabrication, the absorption characteristics of these metal-based MMAs remain non-tunable, significantly limiting their practical applicability. Consequently, there has been growing interest in developing actively tunable MMAs featuring materials with adjustable conductivity to achieve dynamic control of absorption performance.

Graphene, a single layer of carbon atoms arranged in a hexagonal honeycomb lattice through sp^2^ hybridization, has emerged as a pivotal material for the development of advanced terahertz (THz) devices in recent years [26,27,28]. Owing to its exceptional electrical, mechanical, and chemical properties, graphene exhibits high flexibility, stability, and minimal electron mobility loss. Furthermore, its surface conductivity can be dynamically tuned by adjusting the Fermi energy via an externally applied DC voltage, enabling precise control of its electromagnetic response. These distinctive characteristics make graphene an ideal platform for tunable and high-performance THz metamaterials and optoelectronic applications [29,30]. In addition to electrical modulation, graphene-based THz absorbers may be modulated mechanically [31], optically [32], thermally [33], and magnetically [34]. Of these, electrical and mechanical modulations are the most useful and practical.

In this research, a graphene-based broad-band tunable terahertz (THz) metamaterial absorber is proposed, designed to overcome the limitations of conventional narrowband absorbers. The structure consists of a metallic back reflector, a dielectric spacer, and a graphene metasurface patterned in an asymmetric graphene resonator geometry. This configuration enables strong coupling between the graphene resonator and the underlying layers, effectively minimizing reflection and producing near-unity absorption across a wide spectral range. Simulation results demonstrate broad-band absorption exceeding 90% in the frequency range of 2.45–6.11 THz, with three pronounced resonant peaks at 2.764 THz, 3.534 THz, and 5.41 THz, achieving maximum absorption efficiencies of 97.26%, 96.96%, and 99.90%, respectively. A detailed parametric study reveals that the absorber maintains high performance for both transverse electric (TE) and transverse magnetic (TM) polarizations, and preserves its absorption stability for incident angles up to 60°, confirming its polarization- and angle-insensitive nature. The enhanced absorption originates from the simultaneous excitation of electric and magnetic resonances within the multilayer system, as verified by impedance matching and field distribution analyses. With its broad bandwidth, excellent tunability, and simple planar configuration, the proposed absorber holds strong potential for terahertz sensing, imaging, stealth, and photonic device applications.

## 2. Design and Simulations

**Figure 1:**
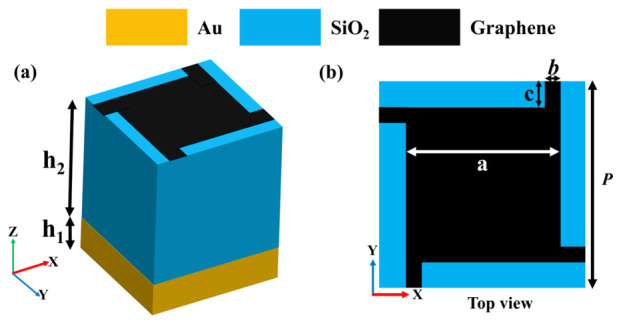
(**a**) Schematic view of THz metamaterial absorber. (**b**) Top view of the proposed absorber.

The proposed absorber consists of a graphene metasurface patterned in an asymmetric graphene resonator geometry placed atop a three-layer structure, as illustrated in Figure 1a. The optimized design was obtained after extensive parametric analysis and material selection to achieve broad-band absorption within the terahertz range. The structure comprises a metallic back reflector (Au), a dielectric spacer, and a monolayer graphene resonator. The gold substrate acts as a perfect reflector, suppressing transmission and ensuring complete absorption of incident waves, characterized by an electrical conductivity of σ = 4.56 × 10^7^ S/m [35]. The intermediate dielectric layer is composed of SiO_2_, chosen for its stability and low loss characteristics, with a relative permittivity of ε_r_ = 3.9 [36,37]. The graphene layer, with an effective thickness of 1 nm, is patterned into an asymmetric resonator, enabling multiple localized plasmonic modes that contribute to broad-band absorption through hybrid resonance coupling. Figure 1b illustrates the geometric dimensions of a single unit cell with optimized parameters: period P = 8 μm, arm length c = 0.9 μm, square patch a = 6.2 μm, arm width b = 0.1 μm, metal thickness $h_{1}\;=\;0.2\;\mu m$, and dielectric thickness $h_{2}\;=\;9.3\;\mu m$. These optimized parameters enable the structure to produce three distinct absorption peaks at 2.764 THz, 3.534 THz, and 5.41 THz, resulting in an overall broad-band absorption exceeding 90% from 2.45 THz to 6.11 THz.

Graphene is a monolayer film composed of closely spaced carbon atoms organized in a hexagonal honeycomb lattice [38]. Both intra- and inter-bands regulate graphene’s conductivity. The conductivity equation is expressed as follows [39,40]:

(1) $$\sigma_{g}(\omega ,\mu ,\Gamma ,T)=\sigma_{intra}+\sigma_{inter}$$

(2) $$\sigma_{intra}=\frac{ie^{2}k_{B}T}{\pi \hbar^{2}\left(\omega +i2\Gamma \right)}\left[\frac{\mu_{c}}{k_{B}T}+2ln(e^{\left(-\frac{\mu_{c}}{k_{B}T}\right)}+1)\right]$$

(3) $$\sigma_{inter}=\frac{{ie}^{2}}{4\pi \hbar }ln\left[\frac{2\left|\mu_{c}\right|-\left(\omega +i2\Gamma \right)\hbar }{2\left|\mu_{c}\right|+\left(\omega +i2\Gamma \right)\hbar }\right]$$

where T is the temperature in Kelvin (T = 300 K), $\mu_{c}$ is the chemical potential, ℏ is the reduced Planck constant, ω is the angular frequency, Γ=1/2τ is the scattering rate, e is the elementary charge, and $k_{B}$ is the Boltzmann constant.

The following relationship can be used to calculate the electrical conductivity ($\sigma_{g}$) of graphene [41,42]:

(4) $$\sigma_{intra}=\frac{e^{2}\mu_{c}}{\pi \hbar^{2}}\frac{i}{\left(\omega +i/\tau \right)}$$

The electrical conductivity of graphene can be dynamically tuned by adjusting its Fermi energy level $\left(E_{F}\right)$, enabling control over its plasmonic response in the terahertz regime. In this work, the Fermi level is set to 1 eV, which corresponds to the optimized condition, yielding the highest overall absorption efficiency for the proposed structure. Parametric analyses confirm that this Fermi energy provides the most balanced performance in terms of resonance strength and bandwidth. The carrier relaxation time (τ) of graphene, representing the average time between electron scattering events, is fixed at 0.1 ps throughout the simulations. A relaxation time of this magnitude allows graphene to respond rapidly to incident electromagnetic excitation, thereby supporting strong and broad-band absorption characteristics. These parameter selections are consistent with experimentally achievable values for high-quality graphene films used in terahertz and optoelectronic applications. The frequency-domain solver in CST Microwave Studio is employed to perform theoretical and numerical analyses of the proposed graphene-based metamaterial absorber, enabling the evaluation of both the near-field distribution and the absorption spectrum, A(ω). To accurately model the periodic nature of the structure, periodic boundary conditions are applied along the x and y directions, while an open (add space) boundary condition is imposed along the z-axis to emulate free-space propagation. The incident electromagnetic wave propagates along the negative z-axis, with the electric field polarized along the x-axis and the magnetic field oriented along the y-axis. The absorption characteristics of the proposed structure are evaluated using the relation *A*(ω)=1−R(ω)−T(ω), where A(ω) represents the absorption rate, $R(\omega )={\left|S_{11}\right|}^{2}$ denotes the reflection, and $T(\omega )={\left|S_{21}\right|}^{2}$ denotes the transmission. The reflection coefficient $\left|S_{11}\right|$ and transmission coefficient $\left|S_{21}\right|$ are obtained from CST Microwave Studio simulations. Since the gold (Au) ground plane located at the bottom of the absorber has a thickness much greater than the skin depth of the incident terahertz waves, the transmission becomes negligible. Consequently, the absorption relation simplifies to A(ω)=1−R(ω), implying that perfect absorption occurs when the reflection coefficient $\left|S_{11}\right|$ approaches zero.

## 3. Results and Discussion

**Figure 2:**
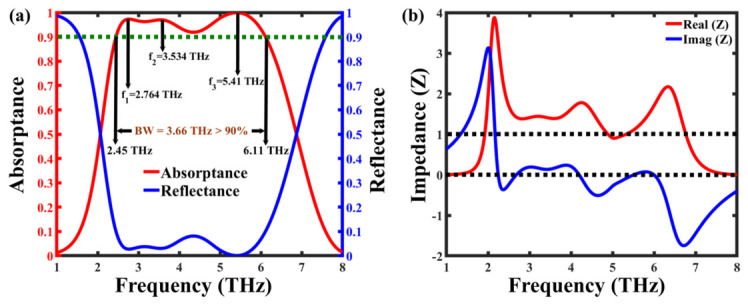
(**a**) Simulated absorption (red) and reflection (blue) spectra of the proposed graphene-based terahertz metamaterial absorber. (**b**) Normalized real and imaginary parts of the effective impedance showing Re(Z) ≈ 1 and Im(Z) ≈ 0 at the resonance frequencies, confirming excellent impedance matching and near-perfect absorption.

The absorption and reflection spectra of the proposed graphene-based terahertz metamaterial absorber are depicted in Figure 2a for normal incidence at a fixed graphene Fermi level of 1 eV. The red solid curve represents the absorptance, while the blue solid curve corresponds to the reflectance. Due to the continuous gold ground plane on the rear side of the structure, transmission is completely suppressed (T(ω) ≈ 0), ensuring that the incident electromagnetic energy is either absorbed or reflected. The absorber exhibits a broad-band absorption exceeding 90% across the frequency range of 2.45 THz to 6.11 THz, corresponding to an effective bandwidth of 3.66 THz. Within this range, three distinct resonance peaks are observed at 2.764 THz, 3.534 THz, and 5.41 THz, achieving maximum absorption efficiencies of 97.26%, 96.96%, and 99.90%, respectively.

The impedance matching theory further explains the broad-band absorption mechanism. For maximum absorption to occur, the effective impedance of the metamaterial (Z) must closely match the impedance of free space $\left(Z_{0}\;=\;377\;\Omega \right)$, thereby minimizing reflection losses. As shown in Figure 2b, the real part of the normalized impedance (Re(Z)) approaches unity, and the imaginary part (Im(Z)) tends toward zero at the resonance frequencies, confirming near-perfect impedance matching. This condition leads to maximum absorption and negligible reflection, while the presence of the metallic backplane eliminates transmission, causing the incident electromagnetic energy to be fully dissipated as heat within the absorber layers. The relative impedance is calculated using the following relation:

(5) $$Z_{r}\left(\omega \right)=\frac{Z\left(\omega \right)}{Z_{0}}=\sqrt{\frac{{\left(1+S_{11}\left(\omega \right)\right)}^{2}-S_{21}^{2}\left(\omega \right)}{{\left(1-S_{11}\left(\omega \right)\right)}^{2}-S_{21}^{2}\left(\omega \right)}}$$

where $S_{11}$ and $S_{21}$ denote the reflection and transmission coefficients, respectively. The strong agreement between the impedance-matching results and the absorption spectrum validates that the broad-band and high-efficiency absorption arises from the synergistic coupling of electric and magnetic resonances within the multilayer configuration.

**Figure 3:**
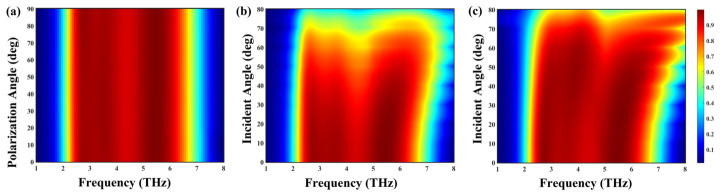
(**a**) Absorption spectra as a function of polarization angle (0–90°), demonstrating polarization insensitivity. (**b**,**c**) Absorption spectra as a function of incident angle for TE and TM polarizations, respectively.

The polarization and incident-angle dependence of the absorber is analyzed to evaluate its robustness under varying illumination conditions, as presented in Figure 3. In Figure 3a, the color map of frequency versus polarization angle shows that the absorption response remains nearly constant as the polarization angle varies from 0° to 90°, confirming that the absorber is polarization-insensitive. This stability originates from the rotationally symmetric geometry of the asymmetric cross-like graphene pattern, which produces equivalent electromagnetic responses for both TE and TM polarizations. The absorber’s angular performance is further examined in Figure 3b and Figure 3c for TE and TM polarizations, respectively. The results indicate that the broad-band absorption above 90% persists up to an incident angle of 60°, with only a slight decrease in intensity at higher angles. This demonstrates the absorber’s excellent angular tolerance and its capability to maintain high efficiency under oblique incidence. Overall, the proposed absorber exhibits ultra-broad-band, polarization-insensitive, and angle-stable absorption characteristics with near-unity efficiency, confirming its potential for integration into practical terahertz imaging, sensing, stealth, and photonic systems.

**Figure 4:**
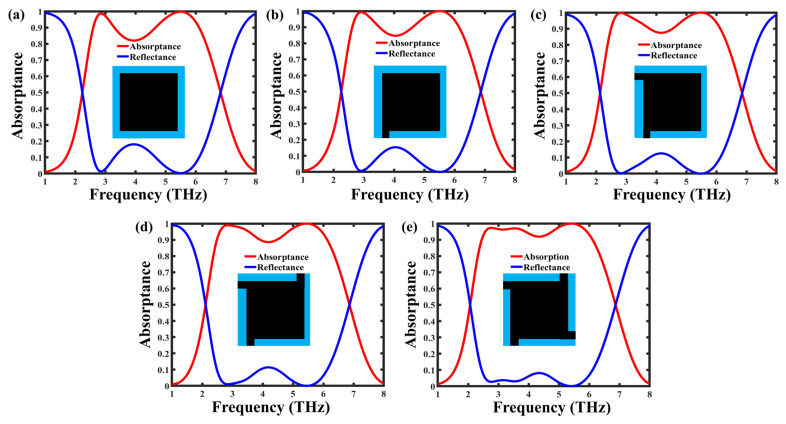
Reflection and absorption spectra of metamaterial absorbers with varying geometric configurations, illustrating the evolution of broad-band absorption with geometric modifications of the graphene resonator. Insets depict the corresponding top-view geometries of each design: (**a**–**c**) initial rectangular and asymmetric configurations exhibiting narrow absorption bands; (**d**) modified cross-like resonator showing partial broad-band response; and (**e**) optimized asymmetric cross-shaped geometry achieving broad-band absorption above 90% with multiple resonance peaks across 2.45–6.11 THz.

To investigate the influence of the graphene resonator geometry on absorption performance, several structural configurations were analyzed, as shown in Figure 4a–e, representing the progressive evolution toward the optimized asymmetric absorber. The initial configurations in Figure 4a–c, consisting of symmetric and partially asymmetric graphene patches, exhibit limited absorption bandwidth with only one or two dominant resonances due to restricted plasmonic mode excitation. As the geometry transitions toward a more asymmetric cross-like structure, additional current paths and coupling regions are introduced, enabling the excitation of multiple non-degenerate localized surface plasmon resonances at different frequencies. This symmetry breaking lifts mode degeneracy and generates several closely spaced resonances associated with different effective resonant lengths. In Figure 4d, the inclusion of offset arms and corner extensions further enhances electromagnetic coupling between adjacent elements, promoting resonance hybridization and spectral overlap. Consequently, the optimized structure in Figure 4e exhibits three distinct resonances and achieves broadband absorption exceeding 90% across the 2.45–6.11 THz range. In addition, the presence of multiple resonances improves impedance matching with free space over a wider frequency range, thereby reducing reflection losses. This progressive analysis clearly demonstrates that the enhanced broadband performance is directly governed by the introduced geometrical asymmetry.

**Figure 5:**
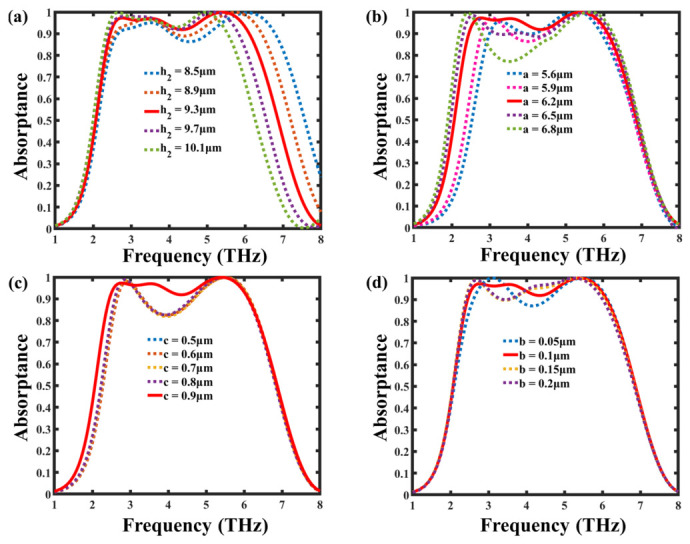
Parametric analysis of the proposed graphene-based terahertz absorber showing the effect of geometric variations on absorption spectra: (**a**) dielectric layer thickness (h_2_), (**b**) arm length (a), (**c**) arm width (c), and (**d**) inter-arm gap (b). The results indicate that the absorber maintains broad-band absorption above 90% across the optimized range, confirming its strong design tolerance and stable resonance behavior.

To investigate the influence of key geometrical parameters on the absorption performance, a comprehensive parametric study was conducted, as illustrated in Figure 5a–d. The parameters examined include the dielectric layer thickness (h_2_), graphene central-square patch (a), arm length (c), and arm width (b). As shown in Figure 5a, the absorber’s bandwidth and resonance strength are highly sensitive to the dielectric thickness. Increasing $h_{2}$ from 8.5 μm to 10.1 μm results in a slight redshift of the resonance frequencies and enhances impedance matching, yielding broader absorption. In Figure 5b, variation in the square patch a between 5.6 μm and 6.8 μm significantly influences the resonant modes, and an increase in the size of the square patch results in a redshift of the resonant frequency and a moderate enhancement in absorption efficiency, which can be attributed to stronger plasmonic coupling and improved resonance confinement within the graphene structure. The effect of arm length c is presented in Figure 5c; increasing c from 0.5 μm to 0.9 μm slightly modifies the bandwidth while maintaining absorption above 80%, indicating good structural tolerance. Similarly, as shown in Figure 5d, the absorber remains stable when the arm width b varies from 0.05 μm to 0.2 μm, demonstrating that the proposed structure sustains robust broad-band absorption even under geometric deviations. These results confirm that the optimized dimensions $\left(h_{2}=9.3\;\mu m,\;a=6.8\;\mu m,\;c=0.9\;\mu m,\;and\;b=0.1\;\mu m\right)$ yield the best broad-band absorption with strong multi-resonant coupling and reliable fabrication tolerance.

**Figure 6:**
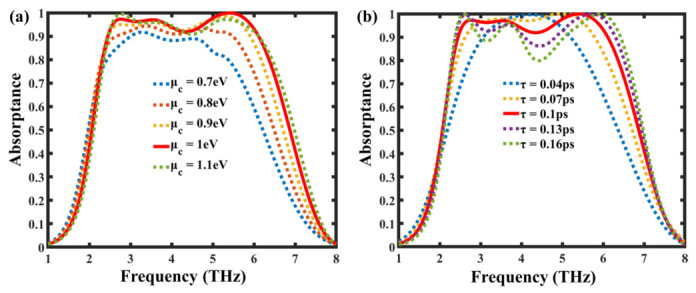
Simulated absorption spectra of the proposed graphene-based terahertz absorber for varying electrical parameters: (**a**) chemical potential $\left(\mu_{c}\right)$ ranging from 0.7 eV to 1.1 eV, showing enhanced absorption and a redshift in resonance peaks with increasing μc; (**b**) carrier relaxation time (τ) ranging from 0.04 ps to 0.16 ps, illustrating the influence of electron scattering on resonance sharpness and absorption bandwidth.

The tunable optical response of the proposed absorber is primarily governed by the electrical properties of graphene, and particularly its chemical potential $\left(\mu_{c}\right)$ and carrier relaxation time (τ). The impact of these parameters on absorption performance is depicted in Figure 6a–b. As shown in Figure 6a, increasing the chemical potential from 0.7 eV to 1.1 eV leads to a gradual enhancement in absorption intensity and a slight redshift of the resonant frequencies. This behavior occurs because a higher chemical potential increases the surface conductivity of graphene, thereby strengthening plasmonic resonance coupling and improving impedance matching across the THz range. In Figure 6b, the relaxation time τ is varied from 0.04 ps to 0.16 ps to evaluate its influence on loss and bandwidth. As τ increases, the absorption peaks become sharper and more pronounced due to reduced carrier scattering and longer electron lifetimes. A relaxation time of 0.1 ps provides an optimal balance between bandwidth and absorption strength, consistent with experimentally achievable values for high-quality graphene films. These findings confirm that the absorber’s spectral characteristics can be dynamically tuned through external biasing, enabling real-time control of absorption properties for applications in reconfigurable terahertz photonic devices and modulators.

**Figure 7:**
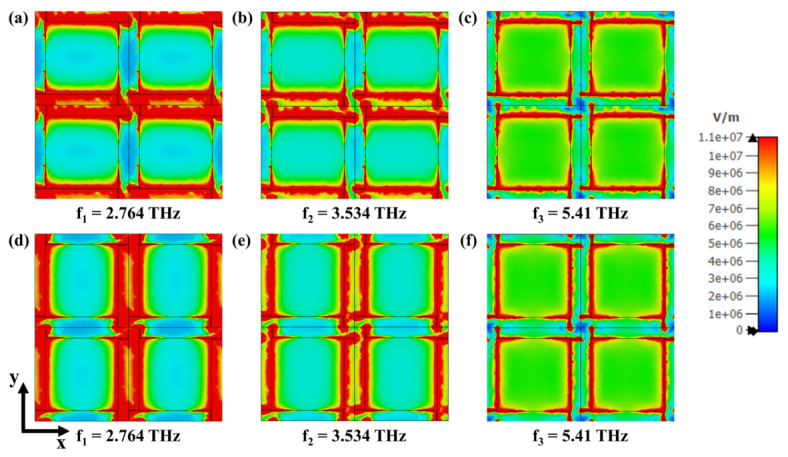
Simulated electric field (|E|) distributions of the proposed graphene-based absorber at three resonance frequencies for TE and TM polarizations. (**a**–**c**) TE modes at $f_{1}\;=\;2.764\;THz$, $f_{2}\;=\;3.534\;THz$, and $f_{3}\;=\;5.41\;THz$, respectively. (**d**–**f**) TM modes at the same frequencies.

To further elucidate the underlying absorption mechanism, the electric field (|E|) distributions of the proposed graphene-based absorber were analyzed for both TE and TM polarizations at the three resonance frequencies, as shown in Figure 7a–f. The top row (Figure 7a–c) corresponds to the TE mode, while the bottom row (Figure 7d–f) represents the TM mode. At the first resonance frequency $\left(f_{1}\;=\;2.764\;THz\right)$, strong electric field confinement occurs along the inner edges of the graphene cross arms, indicating the excitation of localized surface plasmon resonances. At the second resonance $\left(f_{2}\;=\;3.534\;THz\right)$, the field is more uniformly distributed around the arm intersections, suggesting hybrid coupling between adjacent resonant elements. At the third resonance $\left(f_{3}\;=\;5.41\;THz\right)$, the electric field becomes concentrated near the outer corners of the graphene structure, confirming the formation of higher-order plasmonic modes. For both TE and TM polarizations, the spatial field patterns exhibit nearly identical distributions, demonstrating that the absorber maintains polarization-insensitive behavior. These field profiles validate that the broad-band and high-efficiency absorption originates from the multi-resonant plasmonic coupling and strong electromagnetic confinement within the graphene metasurface.

To benchmark the performance of the proposed absorber, a comparative analysis was carried out with previously reported terahertz metamaterial absorbers, as summarized in Table 1. It is evident that the proposed asymmetric cross-shaped graphene absorber achieves the widest absorption bandwidth (3.66 THz) and highest absorption efficiency (≈99.90%) among similar designs. Furthermore, unlike most metallic or single-resonator structures that exhibit narrowband behavior and polarization dependence, the present design offers polarization- and angle-insensitive broad-band absorption with electrical tunability via Fermi energy adjustment. These features demonstrate the superior adaptability and practical potential of the proposed design for integration into next-generation tunable terahertz systems.

Although similar materials are used in prior terahertz absorbers (Table 1), their performance is often limited by symmetric geometries with fewer resonant modes and narrower bandwidths. In contrast, the proposed asymmetric graphene resonator breaks symmetry and enables multiple non-degenerate plasmonic modes. These generate closely spaced resonances whose spectral overlap significantly broadens the absorption bandwidth. The multiple resonances also improve impedance matching over a wider frequency range, reducing reflection losses. These structural advantages account for the enhanced broadband performance of the proposed design.

## 4. Conclusions

In conclusion, a graphene-based broad-band tunable terahertz (THz) metamaterial absorber with an asymmetric graphene configuration has been successfully designed and analyzed. The absorber employs a simple three-layered structure composed of a graphene metasurface, a SiO_2_ dielectric spacer, and a gold reflective substrate, achieving broad-band absorption exceeding 90% within the 2.45–6.11 THz frequency range. Three distinct resonance peaks are observed at 2.764 THz, 3.534 THz, and 5.41 THz, corresponding to near-unity absorption efficiencies of 97.26%, 96.96%, and 99.90%, respectively. The broad-band absorption mechanism was analyzed through impedance matching and electromagnetic field distribution analyses, confirming that the enhanced performance arises from the synergistic coupling of multiple plasmonic resonances within the patterned graphene structure. Furthermore, the absorber exhibits excellent polarization and incident angle insensitivity, maintaining high absorption for both TE and TM polarizations up to 60°. The absorption characteristics can also be dynamically tuned by adjusting the graphene chemical potential and relaxation time, enabling real-time control of resonance strength and bandwidth. These results demonstrate that the proposed asymmetric graphene absorber provides a robust platform for high-performance, tunable THz devices, offering new opportunities for broad-band sensing, imaging, and stealth applications.

## Figures and Tables

**Table 1:** Performance comparison of the proposed absorber with previously reported terahertz absorbers.

Ref.	Absorption Bandwidth	Absorptivity	Material	Tunable	Polarization Insensitivity	Year Published
[4]	3.39 THz	>90%	Graphene-SiO_2_-Au	Yes	Yes	2024
[10]	3 THz	>90%	Graphene-SiO_2_-Au	yes	yes	2024
[30]	3.39 THz	>90%	Graphene-SiO_2_-Au	yes	yes	2025
[43]	1.96 THz	>90%	Graphene-PTFE-Au	Yes	Yes	2025
[44]	3 THz	>90%	Graphene-SiO_2_	yes	yes	2024
[45]	3.10 THz	>90%	Graphene-SiO_2_-Au	yes	yes	2025
[46]	2.47 THz	>90%	Au-Graphene-SiO_2_-Au	yes	yes	2025
[38]	2.7 THz	>90%	VO_2_-TOPAS-Au	yes	yes	2024
[47]	2.61 THz	>90%	Graphene-SiO_2_-Au	yes	yes	2026
[48]	1.98 THz	>90%	Graphene-Rogers-Au	yes	yes	2026
[49]	1.8 THz	>90%	MoS_2_-Graphene-SiO_2_-Si-Au	yes	yes	2026
Our work	3.66 THz	>90%	Graphene-SiO_2_-Au	yes	yes	―

## Data Availability

Data is contained within the article.
